# Oral Lesions in People Living with HIV: The Lining HIV Study

**DOI:** 10.3390/pathogens15070679

**Published:** 2026-06-26

**Authors:** Maria Gavatha, Emmanouil Angelos Rigopoulos, Miranda Alexopoulou, Vasileios Petrakis, Nikoleta Babaka, Olga Tsachouridou, Dimitrios Pilalas, Charis Chari, Alexandra Vorria, Evaggelia Bogosian, Petros Ioannou, Sofia Ioannou, Efstratios Patsatzis, Maria N. Gamaletsou, Andreas Rafail Tzatzimos, Periklis Panagopoulos, Symeon Metallidis, Dimitrios Papazoglou, Konstantinos Tosios, Karolina Akinosoglou

**Affiliations:** 1 Department of Medicine, University of Patras, 26504 Rio, Greece; gavatha.maria@yahoo.com (M.G.); agrigopoulos@gmail.com (E.A.R.); 2 Department of Maxillofacial Surgery, University General Hospital of Patras, 26504 Rio, Greece; malexopoulou@gmail.com; 3 2nd Department of Internal Medicine, University General Hospital Alexandroupolis, Democritus University of Thrace, 68100 Alexandroupolis, Greece; vpetraki@med.duth.gr (V.P.); mpampakanikoleta@gmail.com (N.B.); ppanago@med.duth.gr (P.P.); dpapazog@med.duth.gr (D.P.); 4 Infectious Diseases Unit, 1st Internal Medicine Department, AHEPA University Hospital, School of Medicine, Aristotle University of Thessaloniki, 55436 Thessaloniki, Greece; olgat_med@hotmail.com (O.T.); charis_chari@hotmail.com (C.C.); metallidissimeon@yahoo.gr (S.M.); 5 3rd Department of Internal Medicine, Papageorgiou General Hospital, School of Medicine, Aristotle University of Thessaloniki, 56403 Thessaloniki, Greece; 6 Department of Internal Medicine, University Hospital of Heraklion, 71110 Heraklion, Greece; aleksandravorria@gmail.com (A.V.); bogosiandent@pagni.gr (E.B.);; 7 3rd Department of Internal Medicine and Infectious Diseases Unit, “Korgialenio-Benakio” Red Cross General Hospital of Athens, 11526 Athens, Greece; sofioan7@gmail.com; 8 Department of Dentistry, General Hospital of Athens “Korgialenio-Benakio” Red Cross General Hospital of Athens, 11526 Athens, Greece; patsatzis_stratos@yahoo.gr; 9 Pathophysiology Department, Medical School, National and Kapodistrian University of Athens, 11527 Athens, Greece; magama@med.uoa.gr; 10 Department of Dentistry, Evangelismos General Hospital of Athens, 10676 Athens, Greece; jajimosar@gmail.com; 11 Faculty of Dentistry, National and Kapodistrian University of Athens, 11527 Athens, Greece; ktosios@dent.uoa.gr; 12 Departments of Internal Medicine and Infectious Diseases, University General Hospital of Patras, 26504 Rio, Greece

**Keywords:** HIV, oral health, OHI-S, OHIP-14, oral hygiene, oral health-related quality of life, antiretroviral therapy, oral lesions

## Abstract

Oral manifestations are common in people living with HIV (PLWH) and may affect oral health-related quality of life (OHRQoL), while data from Greece remain limited. This multicenter prospective cohort study evaluated oral health status and OHRQoL among PLWH and explored associations with antiretroviral therapy (ART) and clinical factors. Overall, 370 PLWH from seven referral centers were included. Participants underwent oral examination, with oral hygiene assessed using the Simplified Oral Hygiene Index (OHI-S), and completed the Oral Health Impact Profile-14 (OHIP-14). Statistical analyses were performed using IBM SPSS Statistics v29.0, while multinomial and binary logistic regression identified predictors of oral hygiene status and OHRQoL, respectively. Most participants were male (76.5%), had CD4 counts ≥ 200 cells/μL (95.4%), and were receiving ART (98.6%). Annual dental check-ups, daily tooth brushing, mouthwash use, and dental floss use were reported by 54.1%, 69.5%, 31.9%, and 23.8%, respectively. The median OHI-S score was 2.0 (IQR:1.5–2.7), with 16.9% having poor OHI-S; the median OHIP-14 score was 11 (IQR: 7–15), with 64.4% reporting poor OHRQoL. Male sex was associated with lower odds of poor OHRQoL (OR = 0.377; *p* = 0.006), whereas ART regimen independently predicted poor OHRQoL. These findings support patient-centered oral healthcare within HIV care.

## 1. Introduction

Oral manifestations represent some of the earliest and most important clinical indicators of infection with the Human Immunodeficiency Virus (HIV). These lesions may occur in up to 50% of people living with HIV (PLWH) and in as many as 70–80% of individuals with delayed access to care, particularly those with advanced immunosuppression, i.e., CD4 T-lymphocyte counts < 200 cells/μL, presenting with acquired immunodeficiency syndrome (AIDS) [1]. Oral manifestations associated with HIV infection have historically been classified into three major groups according to the strength of their association with HIV/AIDS and their clinical presentation, based on the classification proposed by the EC-Clearinghouse on Oral Problems Related to HIV Infection and the World Health Organization (WHO) Collaborating Centre for Oral Manifestations of HIV [2]. Group 1 lesions, which are strongly associated with HIV infection, include seven major clinical entities: oral candidiasis, oral hairy leukoplakia, Kaposi sarcoma, linear gingival erythema, necrotizing ulcerative gingivitis, necrotizing ulcerative periodontitis, and non-Hodgkin lymphoma. These lesions often correlate with the degree of immunosuppression and have historically served as clinical indicators of disease progression [3,4]. Group 2 lesions comprise conditions less strongly associated with HIV infection but frequently observed in affected individuals. These include atypical oral ulcerations, salivary gland disease, and infections caused by various viruses such as cytomegalovirus, herpesviruses, human papillomavirus, and varicella-zoster virus [2,4]. Group 3 lesions include rarer conditions that may occur in PLWH, such as oral squamous cell carcinoma and diffuse osteomyelitis, which have been described in association with chronic immune dysfunction or coinfections [2].

The introduction of combination antiretroviral therapy (ART) has dramatically altered the natural history of HIV infection. Since the mid-1990s, ART has resulted in a marked decline in opportunistic infections and HIV-related mortality while substantially improving survival and quality of life among PLWH [5]. Consequently, the prevalence of several HIV-associated oral lesions—particularly oral candidiasis, oral hairy leukoplakia, and Kaposi sarcoma—has significantly decreased, and the reduced occurrence of these lesions is often considered a clinical marker of effective immune reconstitution under ART [6,7]. Nevertheless, ART is also associated with adverse oral effects. Several antiretroviral regimens have been linked to reduced salivary flow and xerostomia, which may disrupt the oral microbiome and facilitate colonization by opportunistic or atypical microorganisms [8]. These alterations in salivary physiology may predispose patients to oral dysfunction and contribute to the development or worsening of oral diseases such as periodontal disease and dental caries [9].

In addition, the COVID-19 pandemic caused substantial disruption in HIV prevention, testing, and care services worldwide. During the past several years, these disruptions have been associated with an increase in delayed HIV diagnoses and a higher proportion of late presenters, potentially once again influencing the epidemiology of HIV-related oral manifestations [10].

On the other hand, the knowledge and attitudes of dentists toward PLWH vary considerably across regions and healthcare systems. Inadequate training and persistent reluctance to provide care for HIV-positive individuals remain concerning issues globally [11]. Importantly, dentists are often among the first healthcare professionals to encounter PLWH, and they therefore play a critical role in the early recognition of oral manifestations suggestive of HIV infection, providing an opportunity for timely referral and linkage to HIV testing and care [12,13]. Despite this, several well-recognized oral indicator conditions for HIV infection are not currently included among the recommended criteria for HIV screening in many professional guidelines [14]. This represents a missed opportunity for earlier diagnosis and intervention.

Oral health is closely linked to both physical and psychosocial well-being; therefore, oral lesions that impair aesthetics, speech, mastication, or general oral function may significantly compromise patients’ health-related quality of life [15]. Achieving the goals of the global HIV response—including the expansion of the UNAIDS “fourth 95” target, which emphasizes quality of life among people receiving HIV care—requires the integration of oral health prevention, screening, and management into comprehensive HIV care [16]. Such an approach necessitates improved awareness and interdisciplinary collaboration between medical and dental professionals. This is particularly important in settings where epidemiological data remain scarce, highlighting the need for studies examining the prevalence, patterns, and determinants of oral lesions among PLWH. The current study aims to evaluate the oral health status of PLWH and investigate its association with disease severity and ART, thereby filling a gap in the current literature in Greece.

## 2. Materials and Methods

### 2.1. Study Design and Participants

This prospective cohort study was conducted between December 2023 and December 2025 in seven referral HIV units located in Patras, Athens, Thessaloniki, Heraklion, and Alexandroupolis, Greece. The participating units were selected based on feasibility, geographic distribution, availability of clinical data, and access to affiliated dental or oral and maxillofacial departments for standardized oral assessment. PLWH who were followed in the participating clinical centers and met the inclusion criteria were consecutively recruited from the collaborating units.

### 2.2. Inclusion and Exclusion Criteria

Participants eligible for the study were individuals with a confirmed diagnosis of HIV infection attending the respective HIV units and who provided written informed consent to participate in the study. Participants were excluded if they were unable or unwilling to provide informed consent, had incomplete clinical or laboratory data, or were unable to undergo oral examination or complete the study questionnaire.

### 2.3. Data Collection

Demographic and clinical data were collected for all participants, including age, sex, clinical history, ART regimen, duration of ART, CD4 cell count, HIV viral load, and hepatitis co-infection status. Additional information regarding oral hygiene practices, dental service utilization, and dietary habits was obtained.

### 2.4. Oral Health and Patient-Related Outcome Assessment Tools

Following recruitment, participants were referred to the respective affiliated Oral and Maxillofacial Surgery or Dental Departments for further clinical evaluation. All participants underwent an oral and dental examination performed by dental professionals to assess oral health status. The examination included the evaluation of dental restorations and the presence of oral lesions.

Oral hygiene status was assessed using the Simplified Oral Hygiene Index (OHI-S). The OHI-S evaluates oral hygiene based on the presence of debris and calculus on selected tooth surfaces, with total scores ranging from 0 to 6, where higher scores indicate poorer oral hygiene. According to standard classification criteria, OHI-S scores were categorized as good (0–1.2), fair (1.3–3.0), and poor (3.1–6.0) [17,18] (Supplementary File S1).

Oral health-related quality of life was assessed using the Oral Health Impact Profile-14 (OHIP-14) questionnaire [19,20]. The OHIP-14 consists of 14 items grouped into seven domains, including functional limitation, physical pain, psychological discomfort, physical disability, psychological disability, social disability, and handicap. Participants rated the frequency of each impact using a five-point Likert scale ranging from 0 (never) to 4 (very often). The total OHIP-14 score ranges from 0 to 56. Scores of 11 or higher indicate poor oral health-related quality of life, whereas scores of 9 or lower indicate good oral health-related quality of life according to previously reported cut-off values [21,22] (Supplementary File S2).

### 2.5. Statistical Analysis

Descriptive statistics were used to summarize the demographic, clinical, and oral health characteristics of the study population. Continuous variables were assessed for normality using the Shapiro–Wilk test and visual inspection of the corresponding Q–Q plots. Based on these assessments, continuous variables were presented as median and interquartile range (IQR). Categorical variables were expressed as frequencies and percentages. Differences among the seven domains of the OHIP-14 were evaluated using the Friedman test.

Participants were classified into three OHI-S categories: good oral hygiene (0–1.2), fair oral hygiene (1.3–3.0), and poor oral hygiene (3.1–6.0) based on reported cut-off values [18]. To identify factors associated with oral hygiene status, multinomial logistic regression analysis was performed, with poor oral hygiene used as the reference category. Demographic, clinical, and HIV-related variables, including sex, age, CD4 count, viral load, and antiretroviral therapy regimen, were included as independent variables. Results were presented as odds ratios (OR) with 95% confidence intervals (CI).

For binary logistic regression analysis, OHIP-14 scores were dichotomized using previously reported cut-off values, with scores ≤ 9 indicating good oral health-related quality of life and scores ≥ 11 indicating poor oral health-related quality of life [21]. Participants with a score of 10 were excluded from this analysis. Binary logistic regression analysis was performed to identify predictors of poor oral health-related quality of life. Both univariate and multivariate analyses were conducted, and results were presented as odds ratios (OR) with 95% confidence intervals (CI).

All tests were two-tailed, and a *p*-value < 0.05 was considered statistically significant. All statistical analyses were performed using IBM SPSS Statistics version 29.0.

## 3. Results

### 3.1. Population Characteristics

A total of 370 participants were included in the study. The demographic and clinical characteristics of the study population are summarized in Table 1. The majority of participants were male (*n* = 283, 76.5%), and most were aged 18–64 years (*n* = 342, 92.4%). The median CD4 count was 676.5 cells/μL (IQR: 459–880), and most participants had CD4 counts ≥ 200 cells/μL (*n* = 353, 95.4%). The median HIV viral load was 20 copies/mL (IQR: 0–20), with 90.8% of participants having an undetectable viral load (<50 copies/mL). HCV RNA was detected in one participant. Eight participants (2.2%) were receiving immunosuppressive therapy, including chemotherapy, infliximab, methotrexate, corticosteroids, trastuzumab, pertuzumab, docetaxel, carboplatin, and a tyrosine kinase inhibitor. The vast majority of participants (*n* = 365, 98.6%) were under ART, with a median treatment duration of 7 years (IQR: 4–10), and the most prevalent regimen was the combination of Nucleoside Reverse Transcriptase Inhibitors (NRTIs) and Integrase Strand Transfer Inhibitors (INSTIs). Other regimens included NRTIs combined with non-nucleoside reverse transcriptase inhibitors (NNRTIs) or protease inhibitors (PIs).

### 3.2. Preventive Dental Care and Dietary Habits

Preventive dental care practices and dietary habits are summarized in Table 2. Data on dental visits were available for 342 participants. The median total number of dental visits was 11 (IQR: 8–18). A total of 200 participants (54.1%) reported attending annual dental check-ups. Daily tooth brushing was reported by 257 participants (69.5%), while mouthwash and dental floss use were reported by 118 (31.9%) and 88 participants (23.8%), respectively. Regarding dietary habits, 199 participants (53.8%) reported daily consumption of sugary foods, while 129 (34.9%) reported consumption two to three times per week, 29 (7.8%) two to three times per month, and 13 (3.5%) reported never consuming sugary foods.

### 3.3. Dental Examination and Oral Lesions

Xerostomia (dry mouth) was reported by 101 participants (27.3%). Among these individuals, 62 (61.4%) presented with mild xerostomia, 37 (36.6%) with moderate xerostomia, and 2 (2.0%) with severe xerostomia. Oral candidiasis was detected in 39 participants (10.6%), whereas the majority of the study population (*n* = 330, 89.2%) showed no signs of candidiasis. Recurrent oral ulcerations were reported in 16 participants (4.3%), while hemorrhagic findings were observed in 101 participants (27.3%). Herpetic stomatitis was detected in 2 participants (0.5%), necrotizing ulcerative gingivitis and necrotizing ulcerative gingivostomatitis were each detected in 1 participant (0.3%), whereas oral hairy leukoplakia was identified in 4 participants (1.1%). Regarding dental restorations, 123 participants (33.2%) had no fillings, 151 (40.8%) had fewer than three fillings, and 96 (25.9%) had more than three fillings. Table 3 presents the distribution of dental restorations and oral lesions in the study population.

Additionally, an exploratory descriptive analysis stratified by sex was performed to further characterize demographic, clinical, dental-care, dietary, and oral-health characteristics among male and female participants. The results of this analysis are provided in Supplementary File S3.

### 3.4. Simplified Oral Hygiene Index (OHI-S) Evaluation

**Figure 1:**
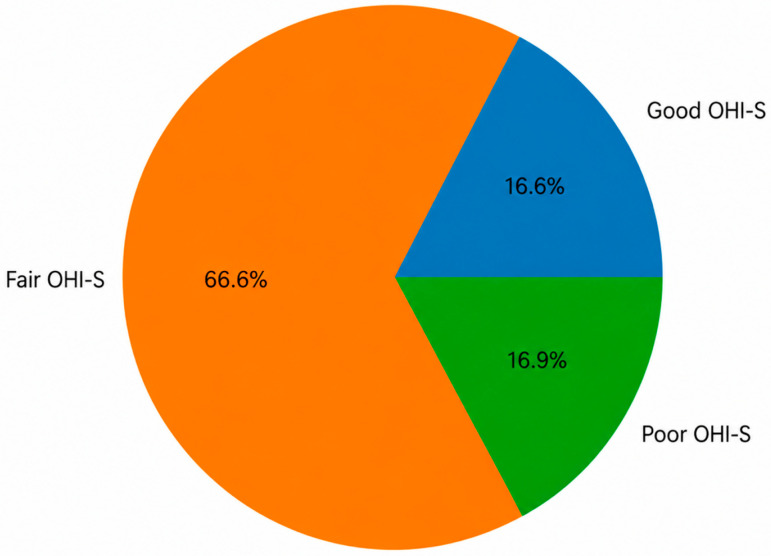
Distribution of OHI-S among participants.

Oral hygiene status assessed using the OHI-S is presented in Figure 1. The median OHI-S score was 2 (IQR: 1.5–2.7) among 332 participants. According to the OHI-S classification, 55 participants (16.6%) had good oral hygiene, 221 (66.6%) had fair oral hygiene, and 56 (16.9%) had poor oral hygiene. (Supplementary Files S3 and S4).

A multinomial logistic regression analysis was performed to identify predictors of oral hygiene status based on the three-category OHI-S classification (Good, Fair, and Poor), with Poor oral hygiene used as the reference category. The overall model did not reach conventional statistical significance (χ^2^(12) = 19.650, *p* = 0.074), with modest explained variance (Cox & Snell R^2^ = 0.068; Nagelkerke R^2^ = 0.083; McFadden R^2^ = 0.040).

In the likelihood ratio tests, sex was the only variable that made a statistically significant contribution to the model (χ^2^(2) = 10.376, *p* = 0.006). Age (χ^2^(2) = 2.994, *p* = 0.224), CD4 count (χ^2^(2) = 0.025, *p* = 0.988), viral load (χ^2^(2) = 0.505, *p* = 0.777), and ART regimen (χ^2^(4) = 6.236, *p* = 0.182) did not reach statistical significance at the overall model level.

Examining the parameter estimates for each outcome category relative to Poor oral hygiene, the following findings emerged. For the Good versus Poor comparison, none of the individual predictors reached statistical significance. Male sex showed a non-significant tendency toward Good oral hygiene compared with female sex (OR = 0.539; 95% CI: 0.134–2.176; *p* = 0.385). Age under 65 years (OR = 0.228; 95% CI: 0.022–2.310; *p* = 0.211), CD4 count above 200 cells/mm^3^ (OR = 1.166; 95% CI: 0.146–9.313; *p* = 0.885), undetectable viral load (OR = 0.574; 95% CI: 0.125–2.645; *p* = 0.476), NRTIs + NNRTIs versus NRTIs + INSTIs (OR = 1.259; 95% CI: 0.166–9.552; *p* = 0.824), and NRTIs + PIs versus NRTIs + INSTIs (OR = 0.458; 95% CI: 0.154–1.362; *p* = 0.160) were all non-significant.

For the Fair versus Poor comparison, male sex was a statistically significant predictor (OR = 0.239; 95% CI: 0.080–0.710; *p* = 0.010), indicating that male participants had significantly lower odds of being classified as Fair rather than Poor compared with female participants. Additionally, participants receiving NRTIs + PIs had significantly lower odds of Fair oral hygiene compared with those receiving NRTIs + INSTIs (OR = 0.372; 95% CI: 0.169–0.819; *p* = 0.014). Age (OR = 0.225; 95% CI: 0.028–1.798; *p* = 0.160), CD4 count (OR = 1.122; 95% CI: 0.215–5.844; *p* = 0.891), viral load (OR = 0.766; 95% CI: 0.221–2.651; *p* = 0.674), and NRTIs + NNRTIs versus NRTIs + INSTIs (OR = 0.685; 95% CI: 0.123–3.805; *p* = 0.665) were not statistically significant.

### 3.5. Oral Health Impact Profile 14 (OHIP-14) Evaluation

**Figure 2:**
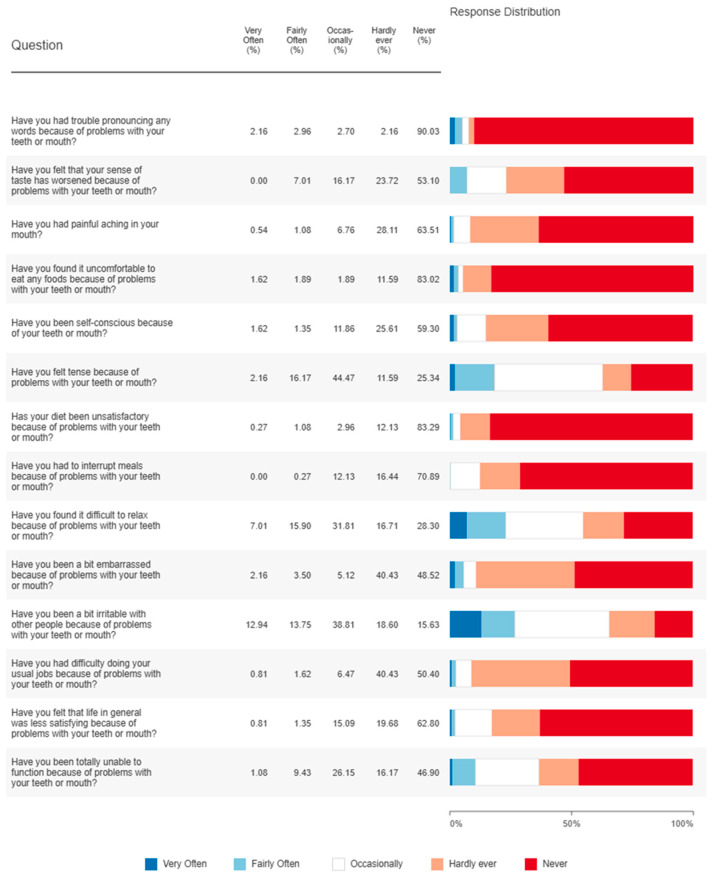
Distribution of responses to the 14 OHIP-14 items. Percentages represent the frequency of each response category (very often, fairly often, occasionally, hardly ever, never) among study participants.

Oral health–related quality of life assessed using the OHIP-14 is presented in Figure 2. The median OHIP-14 score was 11 (IQR: 7–15). Overall, the most frequent response across OHIP-14 items was “never,” indicating that most participants did not report oral health–related problems in their daily activities.

A binary logistic regression analysis was performed to identify predictors of OHRQoL based on the OHIP-14 classification (Good vs. Poor oral health). Based on previously reported cut-off values in the literature, participants with OHIP-14 scores ≤ 9 were classified as having good oral health, while those with scores ≥11 were classified as having poor oral health-related quality of life [21]. Participants with a score of 10 were excluded from the binary analysis. This score represented an intermediate/borderline value that could not be unambiguously assigned to either category according to the applied literature-based cut-off. Consequently, 312 participants were included in the final model, of whom 201 (64.4%) were classified as having poor oral health-related quality of life and 111 (35.6%) as having good oral health.

The model demonstrated good calibration, as indicated by a non-significant Hosmer–Lemeshow test (χ^2^(3) = 0.166, *p* = 0.983). The explained variance was modest (Cox & Snell R^2^ = 0.064; Nagelkerke R^2^ = 0.087), with an overall classification accuracy of 67.0%.

Both univariate and multivariate analyses were performed (Table 4). In the univariate analysis, male sex was significantly associated with lower odds of poor OHRQoL compared with female sex (OR = 0.378; 95% CI: 0.191–0.746; *p* = 0.004). All other variables age under 65 years (OR = 1.919; 95% CI: 0.832–4.424; *p* = 0.121), CD4 count above 200 cells/mm^3^ (OR = 0.649; 95% CI: 0.202–2.088; *p* = 0.465), undetectable viral load below 50 copies/mL (OR = 0.728; 95% CI: 0.292–1.815; *p* = 0.495), HBsAg positivity (OR = 2.222; 95% CI: 0.245–20.129; *p* = 0.466), and the presence of immunodeficiency or neoplasm (OR = 0.693; 95% CI: 0.251–1.914; *p* = 0.477) were not statistically significant at the univariate level. ART regimen was also a statistically significant factor (*p* = 0.017).

In the multivariate analysis, male sex remained significantly associated with lower odds of poor OHRQoL compared with female sex (OR = 0.377; 95% CI: 0.187–0.760; *p* = 0.006). Furthermore, ART regimen remained as a significant independent predictor of poor OHRQoL. Specifically, participants receiving NRTIs + INSTIs had approximately 4.5 times higher odds of poor oral health-related quality of life compared to those receiving NRTIs + NNRTIs (OR = 4.462; 95% CI: 1.470–13.548; *p* = 0.008). Similarly, participants receiving NRTIs + PIs had approximately 4.4 times higher odds compared to the NRTIs + NNRTIs reference group (OR = 4.380; 95% CI: 1.273–15.067; *p* = 0.019). No significant difference was observed between the NRTIs + INSTIs and NRTIs + PIs groups (OR = 1.019; 95% CI: 0.525–1.976; *p* = 0.956), suggesting comparable effects of these two regimens on OHRQoL.

The remaining predictors were not significantly associated with OHIP-14 status in the multivariate model: age under 65 years (OR = 1.873; 95% CI: 0.796–4.407; *p* = 0.151), CD4 count above 200 cells/mm^3^ (OR = 0.791; 95% CI: 0.232–2.703; *p* = 0.709), undetectable viral load (OR = 0.695; 95% CI: 0.265–1.825; *p* = 0.460), HBsAg positivity (OR = 1.988; 95% CI: 0.214–18.467; *p* = 0.546), and the presence of immunodeficiency or neoplasm (OR = 0.596; 95% CI: 0.201–1.768; *p* = 0.351).

**Figure 3:**
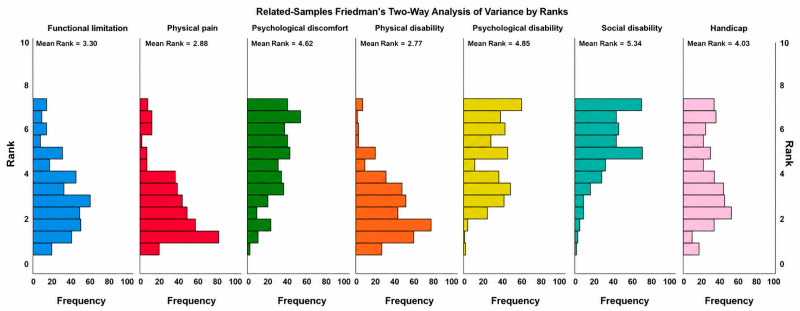
Comparison of mean ranks across the seven OHIP-14 domains using the Friedman test. Higher mean ranks indicate greater impact on oral health-related quality of life.

A Friedman test was performed to compare the distribution of scores across the seven OHIP-14 domains. The analysis demonstrated statistically significant differences among the domains (*p* < 0.001). As illustrated in Figure 3, social disability showed the highest mean rank (5.34), followed by psychological disability (4.85) and psychological discomfort (4.82), indicating that psychosocial aspects of oral health had the greatest impact on quality of life in this cohort.

In contrast, the lowest mean ranks were observed for physical disability (2.77) and physical pain (2.88), suggesting a comparatively smaller impact of physical symptoms on daily functioning. Intermediate values were observed for functional limitation (3.30) and handicap (4.03). Overall, these findings indicate that psychosocial domains of oral health-related quality of life were more strongly affected than the physical domains.

## 4. Discussion

This is the first multicenter study examining oral health in PLWH in Greece. We found that oral hygiene practices and OHRQoL were suboptimal in a substantial proportion of the study population. Although more than half of participants reported annual dental check-ups and the majority practiced daily tooth brushing, the use of adjunctive oral hygiene measures such as mouthwash and dental floss remained limited. Objective oral hygiene assessment demonstrated predominantly fair OHI-S scores, while nearly one in six participants exhibited poor oral hygiene status. Furthermore, almost two-thirds of participants reported impaired OHRQoL, with psychosocial dimensions being the most adversely affected domains. In multivariable analysis, male sex was independently associated with lower odds of poor OHRQoL, whereas ART regimen emerged as an independent predictor of impaired OHRQoL.

Xerostomia and hemorrhagic oral manifestations were relatively common in the study population, affecting more than one-quarter of participants, although most xerostomia cases were mild in severity. Oral candidiasis was identified in approximately one in ten participants, whereas other HIV-associated oral lesions, including recurrent oral ulcerations, oral hairy leukoplakia, herpetic stomatitis, and necrotizing ulcerative lesions, were infrequent. Oral manifestations are early and important clinical indicators of HIV infection, occurring in up to 50% of patients with HIV and 80% of those with AIDS, with seven cardinal lesions internationally recognized as strongly associated with disease progression. A systematic review analyzing 33 studies found that oral lesions were reported in 39% of articles as indicators of HIV disease severity [23]. The seven cardinal lesions—oral candidiasis, hairy leukoplakia, Kaposi sarcoma, linear gingival erythema, necrotizing ulcerative gingivitis, necrotizing ulcerative periodontitis, and non-Hodgkin lymphoma—are seen in both developed and developing countries [24]. A clinical study of 454 patients with HIV found specific lesions strongly predicted severe immunosuppression: major aphthous ulcers showed 100% predictive value for CD4 counts below 200 cells/mm^3^, while necrotizing ulcerative periodontitis showed 95.1% predictive value [3]. These findings establish oral manifestations as reliable surrogate markers for disease progression and immune status, but also reflect the impact of contemporary HIV care and widespread ART use in limiting the prevalence of severe opportunistic oral manifestations.

In our study PLWH generally reported daily tooth brushing but demonstrated poor overall oral health habits, particularly regarding dental visits and fluoride toothpaste use, with a high prevalence of oral lesions. Evidence across multiple studies (n = 4500+ participants) shows mixed patterns. A Tanzanian case–control study of 898 individuals found only 18% of HIV-positive patients had good oral health behaviors (fluoridated toothpaste use and dental visits) [25]. In Nigeria, 71.6% of 134 HIV-positive patients had never visited a dentist [26]. However, daily mouth cleaning was common; 82% of 270 Lesotho patients cleaned their mouths daily, and 77.6% of Nigerian patients brushed their teeth once daily [26,27]. As expected, poor oral hygiene habits were significantly associated with lesion development. Oral lesions were prevalent in 73% of Lesotho patients and 75% of Indian patients (*n* = 126) exhibiting oral mucosal lesions, with candidiasis most common [27].

In our cohort, a considerable proportion of participants demonstrated evidence of prior dental disease burden, with two-thirds presenting at least one dental filling and approximately one-quarter having more than three restorations, suggesting substantial cumulative dental treatment needs within this population. The majority of participants demonstrated fair oral hygiene status according to OHI-S classification, while approximately one-sixth exhibited poor oral hygiene. Although direct comparison with HIV-negative individuals was not possible because the present study did not include a control group, available Greek adult-population data provide useful context for interpreting the OHI-S findings. In a cross-sectional survey of 1218 Greek adults aged 35–44 years, Diamanti et al. assessed oral hygiene using the OHI-S and reported that 52.1% of participants had good oral hygiene, 38.1% fair oral hygiene, and 9.8% poor oral hygiene. In our cohort of PLWH, the corresponding proportion classified as having poor oral hygiene was 16.9%, suggesting a higher frequency of poor oral hygiene status [28]. However, this comparison should be presented cautiously because the reference study included a specific age group from the general adult population, whereas the present cohort consisted of PLWH followed in HIV referral units [28]. Beyond this descriptive comparison, we further explored factors associated with OHI-S classification in the present cohort. The multinomial logistic regression model did not achieve conventional statistical significance overall and demonstrated only modest explanatory capacity; sex emerged as the only variable significantly contributing to oral hygiene classification. Specifically, male sex was associated with significantly lower odds of being categorized as having fair rather than poor oral hygiene compared with female sex.

PLWH face substantial barriers to dental care, including discrimination, cost, and access limitations, with nearly half reporting unmet oral health needs. Evidence is robust across multiple studies. In a study of 2469 PLWH, Fox et al. found that 52.4% had not seen a dentist in over two years and 48.2% reported unmet oral health needs [29]. In the same cohort, Yves Jeanty et al. identified cost, access, and fear as top barriers [30]. Smaller reports have documented discrimination, noting that 58.8% had difficulty registering with dentists versus 18.2% of controls, with 6.2 to even 15% being refused treatment due to HIV status [31,32]. In the context of our findings, potential sex differences may show up differently depending on whether the outcome is care use, unmet need, or oral lesions. One utilization study in Kampala reported that being female (PR=1.16, 95% CI 1.02-1.32) was among the factors associated with not using oral health services [33]. On the other hand, a Brazilian retrospective study found the opposite pattern for oral disease burden, reporting that oral manifestations are less prevalent in females than in males, particularly oral hairy leukoplakia [34]. However, Campisi et al. reported higher overall oral lesion prevalence in women (56.5% vs. 45.5%), particularly oral candidiasis [35]. This discrepancy may reflect differences in CD4 counts between genders across diverse populations.

Even though ART significantly reduces HIV-related oral opportunistic infections, it does not eliminate all oral health complications, with some conditions persisting or emerging despite treatment. The evidence is substantial but nuanced. Tappuni et al. found that 89 HIV patients on combination ART (dual or triple therapy) had significantly fewer oral manifestations than 195 untreated patients [36]. Similarly, Rao et al. demonstrated that oral lesions decreased significantly in 103 patients after ART initiation, correlating with CD4 recovery [37]. However, complications still persist. A recent study observing 498 Australian patients on ART showed increased linear gingival erythema, angular cheilitis, and xerostomia compared to non-ART groups [38]. Patients on long-term ART had greater risks of oral lesions and reduced salivary flow than those without treatment [39]. A systematic review of 97 studies confirmed oral candidiasis remains prevalent (26.2%) even among ART-treated patients [39].

Participants receiving NRTIs + PIs had significantly lower odds of fair oral hygiene compared with those receiving NRTIs + INSTIs, suggesting a potential association between ART regimen and oral hygiene status. By contrast, age, CD4 count, and viral load were not independently associated with oral hygiene categories. Notably, no individual predictor significantly differentiated participants with good versus poor oral hygiene, indicating that determinants of optimal oral hygiene may be multifactorial and not fully captured by the variables included in the present model.

Protease inhibitors have substantially reduced HIV-associated oral complications but produce direct adverse effects on oral tissues. Patton et al. examined 570 Patients with HIV and found overall oral lesion prevalence decreased from 47.6% to 37.5% with protease inhibitor use, including reductions in hairy leukoplakia (25.8% to 11.4%) and necrotizing periodontal disease (4.8% to 1.7%) [40]. For candidiasis specifically, R. Cauda et al. followed 93 patients over one year, finding oral candidiasis in only 7% of protease inhibitor-treated patients versus 36% of non-treated patients [41]. Dios et al. reported similar findings in 75 patients, where candidiasis episodes dropped from 56% to 9.3% after protease inhibitor initiation [42]. However, protease inhibitors produce adverse oral effects. Porter et al. documented oral symptoms including paraesthesia, taste disturbances, and xerostomia [43]. It is possible that certain protease inhibitors (particularly nelfinavir, lopinavir, and saquinavir) inhibit oral epithelial cell DNA synthesis at clinically relevant concentrations, potentially explaining some adverse effects [44].

NNRTI-based antiretroviral therapy is superior to NRTI+PI regimens for reducing oral candidiasis in HIV patients, though evidence is limited to one major comparative study. Ortega et al. [45] examined 1,595 consecutive Brazilian HIV patients over 17 years, comparing NRTI+PI versus NRTI+NNRTI regimens. The study found that patients on NRTI+PI had a relative risk of 0.8 for oral candidiasis compared to NRTI+NNRTI, with this difference being statistically significant (*p* = 0.004). For hairy leukoplakia, NRTI+PI patients were 0.9 times as likely to present with lesions as NRTI+NNRTI users, though this was not statistically significant (*p* = 0.5).

Evidence on the role of INSTIs and oral health remains limited. A recent cross-sectional study of 209 perinatally HIV-infected youth, with 143 on the same ART for at least one year, found that youth receiving ART containing an integrase inhibitor had a mean decayed teeth score 86% higher than those on ART without an integrase inhibitor, after adjusting for age, lifetime viral load suppression, and CD4 nadir [46]. However, this represents the only study found by these authors specifically examining integrase inhibitors and oral health outcomes. The broader literature confirms that oral health problems are prevalent in PLWH in general, but evidence specific to integrase inhibitors remains scarce.

We found that impaired OHRQoL was highly prevalent in this cohort, with nearly two-thirds of participants classified as having poor OHRQoL according to OHIP-14 criteria. Although the majority of responses across individual OHIP-14 items were “never,” indicating limited day-to-day oral health complaints overall, the cumulative burden of oral health impairment remained substantial. The regression model demonstrated good calibration but only modest explanatory power, suggesting that OHRQoL is influenced by multiple interacting factors beyond the variables included in the present analysis. Additionally, available Greek adult-population data provide useful context for interpreting the OHIP-14 findings. In a cross-sectional study of Greek adults aged 35–44 years using the validated Greek OHIP-14, Papaioannou et al. reported a low overall weighted OHIP-14 score of 1.1 ± 1.9. In contrast, the median OHIP-14 score in our cohort of PLWH was 11, and nearly two-thirds of participants were classified as having poor OHRQoL, suggesting a greater perceived oral health burden [47].

In both univariate and multivariate analyses, male sex was independently associated with significantly lower odds of poor OHRQoL, whereas ART regimen emerged as a significant independent determinant of impaired OHRQoL. Specifically, participants receiving NRTIs + INSTIs or NRTIs + PIs demonstrated approximately fourfold higher odds of poor OHRQoL compared with those receiving NRTIs + NNRTIs, while no significant difference was observed between INSTI- and PI-based regimens. By contrast, age, immunological status, viral suppression, HBsAg positivity, and the presence of immunodeficiency or neoplastic disease were not significantly associated with OHRQoL in the adjusted analysis.

An apparently contrasting pattern was observed regarding sex-related differences in objective oral hygiene status and perceived oral health-related quality of life. Male participants showed a less favorable objective oral hygiene profile, as poor OHI-S was more frequent among males than females (20.6% vs. 5.1%). In contrast, female participants reported a greater subjective burden of oral health impairment, with higher rates of poor OHRQoL according to OHIP-14 compared with males (79.7% vs. 59.3%). These findings should not be interpreted as indicating that poorer oral hygiene is associated with better OHRQoL. Rather, they suggest that clinical oral hygiene status and self-perceived OHRQoL may not always follow the same pattern. This difference may reflect sex-related differences in health perception, symptom appraisal, psychosocial distress, and concern regarding the aesthetic or social aspects of oral health. Therefore, OHI-S and OHIP-14 should be considered complementary measures of oral health in PLWH, rather than measures that capture the same aspect of oral health. Although similar sex-related patterns have been described in non-HIV populations, direct comparisons with HIV-negative individuals should be made with caution because the present study did not include a matched HIV-negative control group [48,49].

Oral health significantly impairs quality of life in HIV-positive individuals, with consistent evidence across multiple studies showing strong associations between oral conditions and both physical and mental health outcomes. A large longitudinal study of 2,864 HIV patients found that each additional oral symptom decreased oral health scores by 3.97 points, and oral health improvements were associated with gains in both mental and physical health [50]. Cross-sectional studies consistently demonstrate high prevalence of oral health impacts; 62.6% of 594 low-income PLWH reported oral impacts in the preceding month [51], while 48.8% of 43 institutionalized patients reported poor OHRQoL [52]. A systematic review of 11 studies identified dental caries and periodontitis as primary contributors to reduced quality of life [53]. Importantly, the domain-specific analysis demonstrated that psychosocial dimensions of oral health exerted the greatest negative impact on quality of life, with social disability, psychological disability, and psychological discomfort ranking highest among OHIP-14 domains. In contrast, physical pain and physical disability were comparatively less affected. These findings suggest that the burden of oral health impairment in this population may extend beyond overt physical symptoms and may predominantly influence psychosocial well-being, social functioning, and self-perception.

A number of limitations of the present study should be acknowledged. First, although this was a multicenter study including participants from seven referral HIV units across Greece, the observational design precludes the establishment of causal relationships between HIV-related parameters, ART regimens, and oral health outcomes. The findings therefore should be interpreted as associations rather than evidence of causality. In addition, participants were recruited from tertiary referral HIV centers, which may limit the generalizability of the results to PLWH with different sociodemographic or healthcare characteristics, hard to access or retain in care, including moving populations or intravenous drug users. Second, although a relatively large cohort was included, several subgroup analyses may have been underpowered, particularly for less frequent oral manifestations and for comparisons between ART regimen categories. This may partly explain why the multinomial logistic regression model for OHI-S classification did not achieve conventional statistical significance overall and demonstrated only modest explanatory capacity. Similarly, the wide CIs observed for some variables suggest limited precision of certain estimates. Residual confounding also cannot be excluded, as potentially relevant variables such as smoking status, alcohol consumption, socioeconomic status, educational level, dietary habits, psychiatric comorbidities, salivary flow measurements, duration of HIV infection, cumulative ART exposure, medication-related xerostomia, and access to dental care were not comprehensively incorporated into the multivariable models. The absence of a matched HIV-negative control group also limited direct comparisons with the general population; therefore, comparisons with available Greek adult-population data were descriptive and should be interpreted cautiously. In addition, sugar-containing food consumption was recorded only as an overall frequency category, without information on the specific types, quantities, or sugar content of foods and beverages consumed. Oral hygiene and dietary habits were self-reported and may therefore be affected by recall or reporting bias, unlike clinical oral findings and OHI-S scores, which were assessed through oral examination. Finally, sexual orientation, men who have sex with men (MSM) status, and route of HIV transmission were not systematically collected or analyzed; therefore, we could not determine whether MSM status confounded or modified the observed sex-related associations with OHI-S or OHRQoL. Third, OHRQoL was assessed using a self-administered questionnaire and therefore remains subject to reporting and perception bias. Although the OHIP-14 is a validated and widely used instrument, subjective psychosocial perceptions may be influenced by cultural, emotional, and social factors beyond objective oral health status. This may partly explain the discrepancy between the predominantly fair oral hygiene scores and the substantial burden of impaired psychosocial OHRQoL observed in the cohort. Furthermore, the dichotomization of OHIP-14 scores and exclusion of participants with intermediate scores may have resulted in some loss of information and statistical power. In addition, oral examinations were performed across multiple centers and by different dental professionals, which may have introduced interobserver variability despite the use of standardized assessment tools. The study also relied primarily on clinical examination without adjunctive microbiological, radiological, or salivary biomarker analyses that could have provided additional mechanistic insight into oral dysbiosis, inflammatory burden, or xerostomia-related pathology in PLWH. Finally, the study population consisted predominantly of virologically suppressed individuals receiving ART, with relatively preserved immunological status. Consequently, the prevalence of severe HIV-associated oral lesions was low, limiting the ability to evaluate associations between advanced immunosuppression and oral manifestations. Nevertheless, this finding may also reflect the evolving contemporary HIV landscape in the ART era, in which psychosocial dimensions of oral health appear to remain clinically relevant despite improved virological control.

## 5. Conclusions

In conclusion, the present multicenter study demonstrates that, although severe HIV-associated oral manifestations are currently uncommon in the contemporary ART era, oral health burden and impaired OHRQoL remain highly prevalent among PLWH in Greece. Our results support the growing recognition that integrated and patient-centered oral healthcare approaches are needed for PLWH. Previous studies have demonstrated that multidisciplinary strategies such as integrated medical–dental care pathways, case management support, and mobile or community-based dental services may improve access to oral healthcare and long-term outcomes among vulnerable HIV populations [54]. In this context, the relatively high prevalence of impaired OHRQoL observed in our cohort suggests that there remains substantial room for improvement in preventive oral healthcare, early intervention, patient education, and psychosocial support services within HIV care frameworks. Future research should focus on longitudinal evaluation of oral health trajectories in PLWH, the impact of contemporary ART regimens on oral and salivary health, and the role of behavioral, socioeconomic, microbiome, and inflammatory factors in shaping oral health outcomes. In addition, interventional studies evaluating integrated oral healthcare models and targeted preventive strategies are warranted to determine whether improved access to comprehensive dental care can translate into meaningful improvements in quality of life and overall well-being for PLWH.

## Figures and Tables

**Table 1:** Population characteristics (*n* = 370).

Characteristic	Value
Demographics	
Sex, male, *n* (%)	283 (76.5)
Age 18–64 years, *n* (%)	342 (92.4)
Age ≥ 65 years, *n* (%)	28 (7.6)
CD4 count	
CD4 (cells/mm^3^), Median (IQR)	676.5 (459–880)
<200 cells/mm^3^, *n* (%)	17 (4.6)
≥200 cells/mm^3^, *n* (%)	353 (95.4)
Viral load	
Viral load (copies/mL), Median (IQR)	20 (0–20)
Undetectable or <50 copies/mL, *n* (%)	335 (90.8)
≥50 copies/mL, *n* (%)	34 (9.2)
Hepatitis status	
HBsAg, *n* (%)	5 (1.4)
Anti-HBs, *n* (%)	252 (68.1)
Anti-HBc-IgM, *n* (%)	0 (0.0)
Anti-HBc-IgG, *n* (%)	48 (13.0)
Anti-HCV, *n* (%)	19 (5.1)
Immunosuppression	
Neoplasia, *n* (%)	12 (3.2)
Immunosuppressive therapy, *n* (%)	8 (2.2)
ART	
Receiving ART, *n* (%)	365 (98.6)
Years of ART, Median (IQR)	7 (4–10)
NRTIs + INSTIs, *n* (%)	277 (80)
NRTIs + PIs, *n* (%)	53 (15.3)
NRTIs + NNRTIs, *n* (%)	16 (4.6)

Abbreviations: *n*: number; Interquartile range; ART: Antiretroviral Therapy; NRTIs: Nucleoside Reverse Transcriptase Inhibitors; INSTIs: Integrase Strand Transfer Inhibitors; NNRTIs: Non-Nucleoside Reverse Transcriptase Inhibitors; PIs: Protease Inhibitors.

**Table 2:** Preventive dental care measures and dietary characteristics.

Characteristic	Value
Dental Service Utilization	
Total number of dental visits, Median (IQR)	11 (8–18)
Annual dental check-up, *n* (%)	200 (54.1)
Oral hygiene habits	
Daily oral hygiene, *n* (%)	257 (69.5)
Use of mouthwash, *n* (%)	118 (31.9)
Use of dental floss, *n* (%)	88 (23.8)
Dietary habits	
Sugar-containing food consumption	
Never, *n* (%)	13 (3.5)
2–3 times/month, *n* (%)	29 (7.8)
2–3 times/week, *n* (%)	129 (34.9)
Daily, *n* (%)	199 (53.8)

Abbreviations: IQR: Interquartile range.

**Table 3:** Dental examination and oral lesions.

Fillings	
None, *n* (%)	123 (33.2)
<3, *n* (%)	151 (40.8)
>3, *n* (%)	96 (25.9)
Oral lesions	
Xerostomia (dry mouth), *n* (%)	101 (27.3)
Mild Xerostomia, *n* (%)	62 (61.4)
Moderate Xerostomia, *n* (%)	37 (36.6)
Severe Xerostomia, *n* (%)	2 (2.0)
Oral candidiasis, *n* (%)	39 (10.6)
Recurrent oral ulcerations, *n* (%)	16 (4.3)
Hemorrhagic findings, *n* (%)	101 (27.3)
Herpetic stomatitis, *n* (%)	2 (0.5)
Necrotizing ulcerative gingivitis, *n* (%)	1 (0.3)
Necrotizing ulcerative gingivostomatitis, *n* (%)	1 (0.3)
Oral hairy leukoplakia, *n* (%)	4 (1.1)

**Table 4:** Binary logistic regression analysis of OHIP-14 outcomes.

Predictors	Univariate OR (95% CI)	*p*-Value (Univariate)	Multivariate OR (95% CI)	*p*-Value (Multivariate)
Sex (male)	0.378 (0.191–0.746)	0.004	0.377 (0.187–0.760)	0.006
Age (<65 years)	1.919 (0.832–4.424)	0.121	1.873 (0.796–4.407)	0.151
CD4 count (>200 cells/mm^3^)	0.649 (0.202–2.088)	0.465	0.791 (0.232–2.703)	0.709
Viral load (<50 copies)	0.728 (0.292–1.815)	0.495	0.695 (0.265–1.825)	0.460
HBsAg (positive)	2.222 (0.245–20.192)	0.466	1.988 (0.214–18.467)	0.546
Immunodeficiency/neoplasm (yes)	0.693 (0.251–1.914)	0.477	0.596 (0.201–1.768)	0.351
ART regimen	-	0.017	-	-
NRTIs + INSTIs vs. NRTIs + NNRTIs	-	-	4.462 (1.470–13.548)	0.008
NRTIs + PIs vs. NRTIs + NNRTIs	-	-	4.380 (1.273–15.067)	0.019
NRTIs + INSTIs vs. NRTIs + Pis	-	-	1.019(0.525–1.976)	0.956

Abbreviations: OHIP-14, Oral Health Impact Profile-14; ART, antiretroviral therapy; CD4, cluster of differentiation 4; CI, confidence interval; HBsAg, hepatitis B surface antigen; INSTIs, integrase strand transfer inhibitors; NRTIs, nucleoside reverse transcriptase inhibitors; NNRTIs, non-nucleoside reverse transcriptase inhibitors; OR, odds ratio; PIs, protease inhibitors.

## Data Availability

The data presented in this study are available on reasonable request from the corresponding author. The data are not publicly available due to privacy and ethical restrictions.

## Supplementary Materials

The following supporting information can be downloaded at: https://www.mdpi.com/article/10.3390/pathogens15070679/s1, Supplementary File S1: Assessment of oral hygiene status using the Simplified Oral Hygiene Index (OHI-S); Supplementary File S2: Oral Health Impact Profile-14 (OHIP-14) questionnaire and scoring; Supplementary File S3: Sex-stratified descriptive analysis of demographic, clinical, dental care, dietary, and oral health characteristics among PLWH; Supplementary File S4: Debris and calculus distribution by tooth surface.

## Abbreviations

The following abbreviations are used in this manuscript:

HIV	Human Immunodeficiency Virus
PLWH	People Living with HIV
AIDS	Acquired Immunodeficiency Syndrome
ART	Antiretroviral Therapy
OHRQoL	Oral Health-Related Quality of Life
OHIP-14	Oral Health Impact Profile-14
OHI-S	Simplified Oral Hygiene Index
IQR	Interquartile Range
OR	Odds Ratio
CI	Confidence Interval
HBsAg	Hepatitis B Surface Antigen
Anti-HBs	Antibodies against Hepatitis B Surface Antigen
Anti-HBc	Antibodies against Hepatitis B Core Antigen
Anti-HCV	Antibodies against Hepatitis C Virus
CD4	Cluster of Differentiation 4 T-lymphocytes
NRTIs	Nucleoside Reverse Transcriptase Inhibitors
NNRTIs	Non-Nucleoside Reverse Transcriptase Inhibitors
INSTIs	Integrase Strand Transfer Inhibitors
PIs	Protease Inhibitors
IDU	Infectious Diseases Unit
WHO	World Health Organization
MSM	Men who have Sex with Men
