# Supplementary material for: Oral Lesions in People Living with HIV: The Lining HIV Study
Source: Pathogens. 2026 Jun 26;15(7):679. doi: 10.3390/pathogens15070679 (PMC13414675; doi:10.3390/pathogens15070679)
Supplement: Supplementary file 1 [file pathogens-15-00679-s001.zip › Supplementary File S4 - FINAL.pdf]

Supplementary File S4. Debris and calculus distribution by tooth surface

All participants underwent an oral examination performed by a dental professional, during which the relevant oral sites were systematically assessed.

Debris Distribution by Tooth Surface

Debris distribution data by tooth surface are summarized in Supplementary File S4, Table S1.

For the upper right molar buccal surface, debris was absent in 120 participants, with 127 demonstrating deposits below one-third, 73 between one-third and two-thirds, and 26 with coverage exceeding two-thirds. For the upper left molar buccal surface, debris was absent in 110 participants, with 166 demonstrating deposits below one-third, 39 between one-third and two-thirds, and 33 with coverage exceeding two-thirds.

For the lower right molar lingual surface, debris was absent in 110 participants, with 158 demonstrating deposits below one-third, 50 between one-third and two-thirds, and 23 with coverage exceeding two-thirds. For the lower left molar lingual surface, debris was absent in 90 participants, with 158 demonstrating deposits below one-third, 63 between one-third and two-thirds, and 37 with coverage exceeding two-thirds.

Regarding the anterior teeth, the upper incisor labial surface showed debris absence in 114 participants, with 164 presenting deposits below one-third, 58 between one-third and two-thirds, and 23 with coverage exceeding two-thirds. The lower incisor labial surface showed debris absence in 77 participants, with 201 presenting deposits below one-third, 50 between one-third and two-thirds, and 32 with coverage exceeding two-thirds.

Supplementary File S4, Table S1. Debris Distribution by Tooth Surface

Tooth Surface	No Debris, n (%)	<1/3 of the tooth surface, n (%)	1/3–2/3 of the tooth surface, n (%)	>2/3 of the tooth surface, n (%)
Upper Right Molar (Buccal)	120 (34.7%)	127 (36.7%)	73 (21.1%)	26 (7.5%)
Lower Right Molar (Lingual)	110 (32.3%)	158 (46.3%)	50 (14.7%)	23 (6.7%)

Upper Incisor (Labial)	114 (31.8%)	164 (45.7%)	58 (16.2%)	23 (6.4%)
Lower Incisor (Labial)	77 (21.4%)	201 (55.8%)	50 (13.9%)	32 (8.9%)
Upper Left Molar (Buccal)	110 (31.6%)	166 (47.7%)	39 (11.2%)	33 (9.5%)
Lower Left Molar (Lingual)	90 (25.9%)	158 (45.4%)	63 (18.1%)	37 (10.6%)

Note: Percentages are calculated based on the available number of observations per tooth surface due to missing data.

Calculus Distribution by Tooth Surface

Calculus distribution data by tooth surface are summarized in Supplementary File S4, Table S2.

For the upper right molar buccal surface, calculus was absent in 109 participants, with 151 showing deposits below one-third of the tooth surface, 68 between one-third and two-thirds, and 21 with coverage exceeding two-thirds of the tooth surface. For the upper left molar buccal surface, calculus was absent in 100 participants, with 171 showing deposits below one-third, 65 between one-third and two-thirds, and 14 with coverage exceeding two-thirds.

For the lower right molar lingual surface, calculus was absent in 107 participants, with 139 showing deposits below one-third, 92 between one-third and two-thirds, and 5 with coverage exceeding two-thirds. For the lower left molar lingual surface, calculus was absent in 98 participants, with 151 showing deposits below one-third, 62 between one-third and two-thirds, and 39 with coverage exceeding two-thirds.

Regarding the anterior teeth, the upper incisor labial surface showed absence of calculus in 128 participants, with 149 presenting deposits below one-third, 64 between one-third and two-thirds, and 20 with coverage exceeding two-thirds. The lower incisor labial surface showed the absence of calculus in 84 participants, with 174 presenting deposits below one-third, 61 between one-third and two-thirds, and 41 with coverage exceeding two-thirds.

Supplementary File S4, Table S2. Calculus Distribution by Tooth Surface

Tooth Surface	No Calculus, n (%)	<1/3 of the tooth surface, n (%)	1/3–2/3 of the tooth surface, n (%)	>2/3 of the tooth surface, n (%)
Upper Right Molar (Buccal)	109 (31.2%)	151 (43.3%)	68 (19.5%)	21 (6.0%)
Lower Right Molar (Lingual)	107 (31.2%)	139 (40.5%)	92 (26.8%)	5 (1.5%)

Upper Incisor (Labial)	128 (35.5%)	149 (41.3%)	64 (17.7%)	20 (5.5%)
Lower Incisor (Labial)	84 (23.3%)	174 (48.3%)	61 (16.9%)	41 (11.4%)
Upper Left Molar (Buccal)	100 (28.6%)	171 (48.9%)	65 (18.6%)	14 (4.0%)
Lower Left Molar (Lingual)	98 (28.0%)	151 (43.1%)	62 (17.7%)	39 (11.1%)

Note: Percentages are calculated based on the available number of observations per tooth surface due to missing data.
