# Supplementary material for: Oral Lesions in People Living with HIV: The Lining HIV Study
Source: Pathogens. 2026 Jun 26;15(7):679. doi: 10.3390/pathogens15070679 (PMC13414675; doi:10.3390/pathogens15070679)

Supplementary File S1. Assessment of Oral Hygiene Status using the Simplified Oral Hygiene Index (OHI-S)

The classification of oral hygiene status was performed using the Simplified Oral Hygiene Index (OHI-S) described by Greene and Vermillion (1964) [17].

The oral examination was performed by trained oral health professionals at the dental or maxillofacial departments of the participating units. Six tooth surfaces were examined: four posterior and two anterior teeth.

In the posterior segment of the dentition, the first fully erupted tooth distal to the second premolar, usually the first molar but occasionally the second or third molar, was selected. The buccal surfaces of the selected maxillary molars (16, 26) and the lingual surfaces of the selected mandibular molars (46, 36) were assessed. In the anterior segment, the labial surfaces of the maxillary right (11) and mandibular left (31) central incisors were examined. In cases of missing teeth, appropriate substitutes were used. The numbering of the examined teeth is illustrated in Supplementary Figure S1.

The OHI-S classification has two components: Debris Index Simplified (DI-S) and Calculus Index Simplified (CI-S) [17]. Oral debris refers to soft foreign material loosely attached to the tooth surface, consisting of mucin, bacteria, and food remnants. In contrast, dental calculus is a hardened deposit formed through the mineralization of inorganic salts, mainly calcium carbonate and calcium phosphate, and contains bacteria, food remnants, and shed epithelial cells [17].

Each of the selected tooth surfaces was scored based on the amount of debris and calculus present, with values ranging from 0 to 3 [17].

For the DI-S, a score of 0 indicates no debris, a score of 1 indicates debris covering up to one-third of the tooth surface, a score of 2 indicates debris covering more than one-third but less than two-thirds of the surface, and a score of 3 indicates debris covering more than two-thirds of the surface [17]. The DI-S was calculated by summing the debris scores of the selected teeth and dividing the total by the number of examined tooth surfaces [17].

For CI-S, a score of 0 indicates no calculus, a score of 1 indicates calculus covering up to one-third of the tooth surface, a score of 2 indicates calculus covering more than one-third but less than two-thirds of the surface, and a score of 3 indicates calculus covering more than two-thirds of the surface [17]. The CI-S was calculated by summing the calculus scores of the selected teeth and dividing the total by the number of examined tooth surfaces [17].

The OHI-S score for each patient was calculated as the sum of the DI-S and CI-S scores [17].

Finally, according to standard classification criteria, OHI-S scores were categorized as good (0–1.2), fair (1.3–3.0), and poor (3.1–6.0) [18].

Supplementary Figure S1. Tooth surfaces examined for the assessment of oral hygiene using the OHI-S.

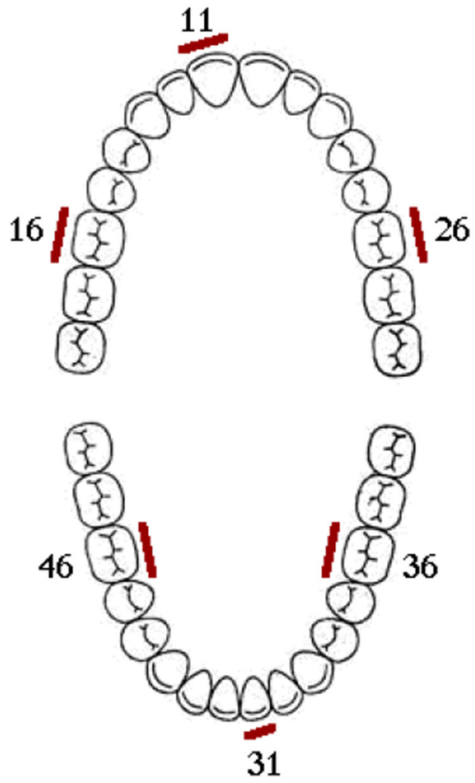

Supplement: Supplementary file 1 [file pathogens-15-00679-s001.zip › Supplementary File S1 -FINAL.pdf]
