# Supplementary material for: Oral Lesions in People Living with HIV: The Lining HIV Study
Source: Pathogens. 2026 Jun 26;15(7):679. doi: 10.3390/pathogens15070679 (PMC13414675; doi:10.3390/pathogens15070679)
Supplement: Supplementary file 1 [file pathogens-15-00679-s001.zip › Supplementary File S2 - FINAL.pdf]

Supplementary File S2. Oral Health Impact Profile-14 (OHIP-14) questionnaire and scoring

Oral health-related quality of life was assessed using the validated Greek version of the Oral Health Impact Profile-14 (OHIP-14). The original Oral Health Impact Profile was developed by Slade and Spencer [19], while the OHIP-14 is widely used as a short-form instrument for assessing oral health-related quality of life [20].

Patients completed the questionnaire anonymously during their oral health assessment.

The OHIP-14 is divided into seven conceptual domains, each represented by two items. The seven domains are the following: functional limitation, physical pain, psychological discomfort, physical disability, psychological disability, social disability, and handicap [19,20].

Responses to each item were recorded on a five-point Likert scale, where 0 = never, 1 = hardly ever, 2 = occasionally, 3 = fairly often, and 4 = very often. The total OHIP-14 score is calculated by summing the responses to all 14 items, resulting in a total score ranging from 0 to 56, with higher scores indicating poorer oral health-related quality of life [19,20]. Patients' OHIP-14 scores were categorized according to previously reported cut-off values, whereby scores ≤9 were classified as indicating good oral health-related quality of life, and scores ≥11 as indicating poor oral health-related quality of life [21,22].

The questionnaire used for assessing oral health-related quality of life (OHIP-14) is presented in Supplementary File S2, Table S1.

Supplementary File S2, Table S1. The 14 items of the validated Greek version of the OHIP-14 that was administered and anonymously completed by study patients during oral health assessment.

OHIP-14 Item	Very often	Fairly often	Occasionally	Hardly ever	Never
1. Have you had trouble pronouncing words because of problems with your teeth or mouth?					
2. Have you felt that your sense of taste has worsened because of problems with your teeth or mouth?					
3. Have you had painful aching in your mouth?					
4. Have you found it uncomfortable to eat any foods because of					

problems with your teeth or mouth?

5. Do your teeth feel strange or unfamiliar to you?

6. Have you felt tense because of problems with your teeth or mouth?

7. Has your diet been unsatisfactory because of problems with your teeth or mouth?

8. Have you had to interrupt meals because of problems with your teeth or mouth?

9. Have you found it difficult to relax because of problems with your teeth or mouth?

10. Have you been a bit embarrassed because of problems with your teeth or mouth?

11. Have you been a bit irritable with other people because of problems with your teeth or mouth?

12. Have you had difficulty doing your usual jobs because of problems with your teeth or mouth?

13. Do you feel that you do not want to go out to eat?

14. Do you feel that you are unable to chew, smile, or speak?	

Abbreviations: OHIP-14: Oral Health Impact Profile-14

Note: Items 5, 13, and 14 were presented using simplified explanatory wording to enhance participant comprehension, while preserving the corresponding OHIP-14 conceptual domains.
