# Supplementary material for: Oral Lesions in People Living with HIV: The Lining HIV Study
Source: Pathogens. 2026 Jun 26;15(7):679. doi: 10.3390/pathogens15070679 (PMC13414675; doi:10.3390/pathogens15070679)
Supplement: Supplementary file 1 [file pathogens-15-00679-s001.zip › Supplementary File S3 - FINAL.pdf]

Supplementary File S3: Sex-stratified descriptive analysis of demographic, clinical, dental care, dietary, and oral health characteristics among PLWH

This supplementary file presents an exploratory descriptive analysis of selected demographic, clinical, dental care, dietary, and oral health characteristics stratified by sex. The analysis was performed further to describe potential differences between male and female participants. Given the unequal distribution of participants by sex and the limited number of female participants, these findings should be interpreted descriptively and not as evidence of independent associations. Categorical variables are presented as numbers and percentages, while continuous variables are presented as medians and interquartile ranges. Chi-square test was used for categorical variables, Fisher's exact test was applied when the expected cell count was <5, and the Mann–Whitney U test was used for continuous variables.

Supplementary file S3- Table S1 summarizes the demographic and clinical characteristics of the study population stratified by sex. Most participants were aged 18–64 years and had CD4 counts ≥200 cells/mm^3, with no significant differences between male and female participants in age distribution, CD4 count, viral load, hepatitis B surface antigen positivity, Anti-HBc-IgG, Anti-HBc-IgM, anti-HCV, immunosuppression-related variables, or ART regimen. Anti-HBs positivity was significantly more frequent among female participants than male participants (80.5% vs. 64.3%, p = 0.007).

Supplementary File S3, Table S1. Population characteristics, by sex (n = 370).

Characteristic	Total (n = 370)	Male (n = 283)	Female (n = 87)	p-value
**Demographics**				
Age 18–64 years, n (%)	342 (92.4)	259 (91.5)	83 (95.4)	0.334
Age ≥65 years, n (%)	28 (7.6)	24 (8.5)	4 (4.6)	
**CD4 count**				
CD4 (cells/mm^3), Median (IQR)	676.5 (459–880)	678.0 (452–866)	669.0 (498–913)	0.542
<200 cells/mm^3, n (%)	17 (4.6)	12 (4.2)	5 (5.7)	0.562*
≥200 cells/mm^3, n (%)	353 (95.4)	271 (95.8)	82 (94.3)	
**Viral load**				
Viral load (copies/mL), Median (IQR)	20 (0–20)	20 (0–20)	20 (20–20)	—
Undetectable (<50 copies/mL), n (%)	335 (90.5)	255 (90.1)	80 (92.0)	0.544
≥50 copies/mL, n (%)	34 (9.2)	28 (9.9)	6 (6.9)	
**Hepatitis status**				

Characteristic	Total (n = 370)	Male (n = 283)	Female (n = 87)	p-value
HBsAg, n (%)	5 (1.4)	4 (1.4)	1 (1.1)	1.000*
Anti-HBs, n (%)	252 (68.1)	182 (64.3)	70 (80.5)	0.007
Anti-HBc-IgM, n (%)	0 (0.0)	0 (0.0)	0 (0.0)	—
Anti-HBc-IgG, n (%)	48 (13.0)	35 (12.4)	13 (14.9)	0.658
Anti-HCV, n (%)	19 (5.1)	17 (6.0)	2 (2.3)	0.266*
**Immunosuppression**				
Neoplasia, n (%)	12 (3.2)	11 (3.9)	1 (1.1)	0.308*
Immunosuppressive therapy, n (%)	8 (2.2)	6 (2.1)	2 (2.3)	1.000*
Antiretroviral therapy (ART)				
Receiving ART, n (%)	360 (98.6)	275 (97.2)	85 (97.7)	1.000*
Years of ART, Median (IQR)	7 (4–10)	7 (4–10)	6 (4–10)	0.889
**ART regimen ****				0.480
NRTIs + INSTIs, n (%)	277 (80.1)	210 (76.4)	67 (78.8)	
NRTIs + PIs, n (%)	53 (15.3)	44 (16.0)	9 (10.6)	
NRTIs + NNRTIs, n (%)	16 (4.6)	13 (4.7)	3 (3.5)	

Abbreviations: IQR: interquartile range; ART: antiretroviral therapy; NRTIs: nucleoside reverse transcriptase inhibitors; INSTIs: integrase strand transfer inhibitors; NNRTIs: non-nucleoside reverse transcriptase inhibitors; PIs: protease inhibitors; HBsAg: hepatitis B surface antigen; Anti-HBs: antibodies against hepatitis B surface antigen; Anti-HBc : antibodies against hepatitis B core antigen; Anti-HCV: antibodies against hepatitis C virus.

Note: Chi-square test was used for categorical variables and Mann–Whitney U test for continuous variables.

*Fisher's exact test was used when the expected cell count was <5.
** For the ART regimen, percentages were calculated among participants receiving ART (n = 360), and the p-value refers to the comparison across all three regimen categories.

Supplementary File S3- Table S2 presents preventive dental care measures, oral hygiene habits, and dietary characteristics stratified by sex. Male participants reported annual dental check-ups more frequently than female participants (59.7% vs. 35.6%, p < 0.001), as well as daily tooth brushing (73.1% vs. 57.5%, p = 0.008) and mouthwash use (35.7% vs. 19.5%, p = 0.007). The overall frequency of sugar-containing food consumption differed significantly by sex (p = 0.001), with daily consumption being more frequently reported by male participants, whereas female participants more frequently reported consumption 2–3 times per week. Total dental visits and dental floss use did not differ significantly between male and female participants.

Supplementary File S3, Table S2. Preventive dental care measures and dietary characteristics, by sex (n = 370).

Characteristic	Total (n = 370)	Male (n = 283)	Female (n = 87)	p-value
**Dental service utilization**				
Total dental visits, Median (IQR)	11 (8–18)	11 (8–18)	11 (8–19)	0.774
Annual dental check-up, n (%)	200 (54.1)	169 (59.7)	31 (35.6)	<0.001
**Oral hygiene habits**				
Daily tooth brushing, n (%)	257 (69.5)	207 (73.1)	50 (57.5)	0.008
Mouthwash use, n (%)	118 (31.9)	101 (35.7)	17 (19.5)	0.007
Dental floss use, n (%)	88 (23.8)	69 (24.4)	19 (21.8)	0.731
**Dietary habits**				
Sugar-containing food consumption				
Overall frequency *				0.001
Never, n (%)	13 (3.5)	12 (4.2)	1 (1.1)	
2–3 times/month, n (%)	29 (7.8)	22 (7.8)	7 (8.0)	
2–3 times/week, n (%)	129 (34.9)	84 (29.7)	45 (51.7)	
Daily, n (%)	199 (53.8)	165 (58.3)	34 (39.1)	

Abbreviations: IQR: interquartile range.

Note: Chi-square test was used for categorical variables and Mann–Whitney U test for continuous variables.

*For sugar-containing food consumption, the p-value refers to the comparison across all four frequency categories.

Supplementary File S3, Table S3 summarizes dental examination findings and oral lesions stratified by sex. The distribution of dental restorations did not differ significantly between male and female participants. Xerostomia and xerostomia severity did not differ significantly between male and female participants. Oral candidiasis was more frequently observed among female participants than male participants (17.2% vs. 8.5%, p = 0.034). No significant sex-related differences were observed for the remaining oral lesions, including recurrent oral ulcerations, hemorrhagic findings, herpetic stomatitis, necrotizing ulcerative gingivitis, necrotizing ulcerative gingivostomatitis, or oral hairy leukoplakia.

Supplementary File S3, Table S3. Dental examination and oral lesions, by sex (n = 370).

Characteristic	Total (n = 370)	Male (n = 283)	Female (n = 87)	p-value
**Dental restorations**				
Fillings **				0.097
None, n (%)	123 (33.2)	88 (31.1)	35 (40.2)	
<3, n (%)	151 (40.8)	124 (43.8)	27 (31.0)	
>3, n (%)	96 (25.9)	71 (25.1)	25 (28.7)	
**Oral lesions**				
Xerostomia (dry mouth), n (%)	101 (27.3)	82 (29.0)	19 (21.8)	0.235
Severity ***				0.501
Mild, n (%)	62 (62.0)	50 (61.7)	12 (63.2)	
Moderate, n (%)	36 (36.0)	30 (37.0)	6 (31.6)	
Severe, n (%)	2 (2.0)	1 (1.2)	1 (5.3)	
Oral candidiasis, n (%)	39 (10.6)	24 (8.5)	15 (17.2)	0.034
Recurrent oral ulcerations, n (%)	16 (4.3)	13 (4.6)	3 (3.4)	0.772 *
Hemorrhagic findings, n (%)	101 (27.3)	74 (26.1)	27 (31.0)	0.449
Herpetic stomatitis, n (%)	2 (0.5)	1 (0.4)	1 (1.1)	0.415 *
Necrotizing ulcerative gingivitis, n (%)	1 (0.3)	1 (0.4)	0 (0.0)	1.000 *
Necrotizing ulcerative gingivostomatitis, n (%)	1 (0.3)	1 (0.4)	0 (0.0)	1.000 *
Oral hairy leukoplakia, n (%)	4 (1.1)	4 (1.4)	0 (0.0)	0.577 *

Note: Chi-square test was used unless otherwise indicated. * Fisher's exact test was used when the expected cell count was <5. ** For fillings, the p-value refers to the comparison across all three filling categories. *** For xerostomia severity, the p-value refers to the comparison across severity categories among participants with xerostomia and valid severity data (n = 100).

Supplementary File S3, Table S4 summarizes OHI-S classification and OHRQoL outcomes stratified by sex. Poor OHI-S was more frequent among male participants than female participants (20.6% vs. 5.1%, p = 0.001). In contrast, poor OHRQoL according to OHIP-14 was more frequently reported by female participants than male participants (79.7% vs. 59.3%, p = 0.003).

Supplementary File S3, Table S4: OHI-S classification and OHIP-14 outcomes, by sex.

Characteristic	Male	Female	p-value
**Poor OHI-S, n/N (%)**	52/253 (20.6)	4/79 (5.1)	0.001
**Poor OHRQoL (OHIP-14), n/N (%)**	159/268 (59.3%)	55/69 (79.7)	0.003

Abbreviations: OHI-S; Simplified Oral Hygiene Index, OHRQoL; Oral health-related Quality of Life, OHIP-14; Oral Health Impact Profile-14

Note: Data are presented as n/N (%), where N represents the available number of observations for each variable. Denominators differ across variables due to missing data
